# Supplementary material for: MetaRibo-Seq measures translation in microbiomes
Source: Nat Commun. 2020 Jun 29;11:3268. doi: 10.1038/s41467-020-17081-z (PMC7324362; doi:10.1038/s41467-020-17081-z)
Supplement: Supplementary file 10 — Supplementary Data 7 [file 41467_2020_17081_MOESM10_ESM.zip › File2/Confidence_High_Taxonomy/63895_out.krona.html]

Javascript must be enabled to view this page.

members
magnitude
magnitudeUnassigned
count
unassigned
taxon
rank

63895\_out

48

2
48
superkingdom

1239
phylum
48

48
class
186801

48
order
186802


SRS014736\_contig\_number\_contig-100\_2252.45573SRS015431\_contig\_number\_80456SRS019685\_contig\_number\_contig-100\_196.194730SRS042284\_contig\_number\_contig-100\_2003.26406
186803
4
family
43

841
5
genus

1262948
1
species

SRS012969\_contig\_number\_16789

2293128

SRS015217\_contig\_number\_30945SRS148159\_contig\_number\_3584SRS148721\_contig\_number\_56614
3
species


SRS020233\_contig\_number\_contig-100\_574.342921
1
species
301302

14
genus
572511

1907659
1
species

SRS017916\_contig\_number\_contig-100\_6099.101978

species
8

SRS014979\_contig\_number\_26166SRS017521\_contig\_number\_526SRS019068\_contig\_number\_55453SRS019910\_contig\_number\_25090SRS049712\_contig\_number\_contig-100\_391.139711SRS065176\_contig\_number\_1288SRS098514\_contig\_number\_contig-100\_953.953SRS1055076\_contig\_number\_contig-100\_134.98972
40520

species
2

SRS149181\_contig\_number\_16464SRS893270\_contig\_number\_4525
1226324

33039

SRS022071\_contig\_number\_37151SRS024549\_contig\_number\_contig-100\_163.48642
2
species

species
1

SRS012273\_contig\_number\_48077
2292990

1
species

SRS049959\_contig\_number\_28317
397290

1
genus
189330

88431
1
species

SRS142712\_contig\_number\_865

33042
genus
1

410072
species
1

SRS023914\_contig\_number\_26442

16
genus
1407607

1150298

SRS013940\_contig\_number\_14534SRS014235\_contig\_number\_contig-100\_1674.277252SRS014613\_contig\_number\_16099SRS014948\_contig\_number\_contig-100\_1144.141438SRS015579\_contig\_number\_contig-100\_386.154897SRS015794\_contig\_number\_contig-100\_1183.91017SRS019161\_contig\_number\_contig-100\_1817.239083SRS050422\_contig\_number\_35238SRS050941\_contig\_number\_contig-100\_348.138156SRS054590\_contig\_number\_contig-100\_1138.1138SRS057717\_contig\_number\_7774SRS097889\_contig\_number\_contig-100\_384.228038SRS104400\_contig\_number\_57077SRS104485\_contig\_number\_contig-100\_101.47785SRS143780\_contig\_number\_33391SRS147139\_contig\_number\_contig-100\_1077.243206
species
16


SRS024075\_contig\_number\_15680
1
species
1898203

2
family
541000

1263
genus
2


SRS144506\_contig\_number\_19333
species
1
46228


SRS144537\_contig\_number\_49579
species
1
657323

2306574
2
species

SRS014459\_contig\_number\_41215SRS015065\_contig\_number\_12731

216572
family
1

1
genus
459786

1
species

SRS147919\_contig\_number\_15760
876091
